# User-Centered Design Approach to Dynamic Clinical Decision Support in Primary Prevention of Cardiovascular Disease

**DOI:** 10.3390/healthcare14182937

**Published:** 2026-09-10

**Authors:** Hannah Husby, Jacqueline Liu, Alexandra Scott, Jiang Li, Qiwen Huang, James Brian Jones, Xiaowei Yan

**Affiliations:** 1 Sutter Health Research Institute, 2121 North California Blvd., Suite 310, Walnut Creek, CA 94596, USA; jacqueline.liu@sutterhealth.org (J.L.); james.jones2@sutterhealth.org (J.B.J.); sherry.yan@sutterhealth.org (X.Y.); 2 Sutter Health Research Institute, 795 El Camino Real, Ames Building, Palo Alto, CA 94301, USA; jiang.li2@sutterhealth.org (J.L.); qiwen.huang@sutterhealth.org (Q.H.)

**Keywords:** cardiovascular disease, primary prevention, user-centered design, qualitative research, surveys

## Abstract

**Highlights:**

**What are the main findings?**

- Primary care assumes responsibility for CVD preventive care but lacks effective tools to manage patients and their unique circumstances.
- Preventive digital tools without up- and downstream support services are insufficient to support preventive care.

**What are the implications of the main findings?**

- Care models supported by integrated tools that target all potential stakeholders can potentially improve prevention.

**Abstract:**

**Background/Objectives**: National guidelines include recommendations for cardiovascular disease (CVD) prevention, but gaps in prevention still exist. Clinical decision support (CDS) tools may be an avenue for delivering preventive care. The goal of this project was to understand how clinicians approach primary prevention of CVD and to determine barriers and facilitators in delivery of primary preventive care with and beyond a CDS tool. **Methods**: A user-centered design approach including a cross-sectional survey and qualitative interviews was deployed in a health system in Central and Northern California. Clinician responses to the survey in addition to clinician interviews about perspectives on CVD were analyzed. **Results**: A total of 62 primary care physicians, advanced practice clinicians, and nurse practitioners completed surveys, and 7 primary care clinicians participated in interviews. Primary care clinicians generally agree that preventive care would be more efficient with team-based care. Physician-centered care and CVD preventive care were constricted by limited time, the highly varied nature of primary care, and a lack of standardized and personalized CDS tools. Once patients at risk of CVD are identified, there is also a lack of clarity around resources available to support downstream care for patients. **Conclusions**: The results highlight the challenges in providing CVD prevention for primary care clinicians. A team-based approach, supported by digital CDS tools, can be more effective for ensuring the necessary CVD prevention communication and coordination takes place.

## 1. Introduction

In the United States and worldwide, cardiovascular disease (CVD) is the leading cause of death [1]. The growing burden of cardiovascular risk factors like obesity, diabetes, elevated cholesterol, and poorly implemented prevention strategies [2] are key contributors to the predicted worsening status of cardiovascular disease (CVD) health. The 2019 American College of Cardiology/American Heart Association (ACC/AHA) guidelines recognize the importance of prevention in addressing unfavorable trends in CVD health and provide evidence-based recommendations for individuals without established CVD; the most recent multi-society lipid management guidelines further highlight a combination of CVD predicted risk and other biometrics to classify patient’s risk in order to provide personalized clinical recommendations [3]. These recommendations focus on comprehensive management strategies including risk identification, healthy lifestyle, and pharmacotherapy [4].

Despite updated guidelines that encourage prevention, major gaps exist in effective CVD prevention strategies in primary care [5]. While guidelines provide a good foundation for care, translating guidelines into clinical practice can be difficult [6,7], due to integration into workflow, the need for tools to facilitate risk classification and clinical decisions, and limited time with patients [5,8,9,10].

Health systems have made efforts to develop point-of-care decision support tools to improve assessment and decision-making for CVD preventive care. Clinical decision support (CDS) tools have demonstrated the potential benefit of providing evidence-based and patient specific recommendations at the point of care but experience challenges in adoption and optimization [11]. As an example, the use of CV Wizard, a nonproprietary web-based CDS tool, was associated with significant decreases in CVD risk [5] but experienced low adoption overall in community health centers [12]. An updated version of CV Wizard called Priorities Wizard, improved for supporting CVD and preventive care and launched by a non-physician member of the care team, experienced high healthcare utilization rates [13]. The adoption difference between the two versions of CDS emphasizes that understanding the context [7] such as end-user acceptance, knowledge, and barriers to adoption of decision support tools is critical for successful CDS implementation. Clinician acceptance of CDS can be promoted by involving them in the development process to ensure CDS meet their needs and preferences [14]. The recent advances in artificial intelligence (AI) technology have the potential to assist doctors in comprehensive clinical decisions. However, studies in this area often ignore how tools fit into a doctor’s actual workflow [14] and demonstrate gaps between the (1) ideal care model that can streamline clinical processes, (2) the roles involved in each care delivery step, and (3) real-world fragmented clinical settings with limited resources. Within healthcare operations, CDS design must include technological advancement as well as a consideration of the care continuum, optimized clinical resource sharing, and workflow integration [15,16].

Therefore, the goal of this quality improvement project was to understand (1) what workflow barriers and facilitators do primary care clinicians (PCCs) experience when delivering CVD prevention and management and (2) what are the perceived roles and responsibilities of physicians and non-physician clinicians (nurse practitioners/physician assistants) in delivering and managing CVD prevention in primary care? We employed the principles of human-centered design (HCD) and user-centered design (UCD) as the conceptual framework for our exploration. The principles of HCD include participatory design, ethnography, cocreation, and empathic design [17]. Similarly, UCD is described as a set of methods in which end-users are involved in and influence the design process [17]. We implemented the principles of HCD/UCD in a product preparation phase to understand and observe how clinicians approach CVD management in their day-to-day practice. We share learnings from this exploratory effort to learn how to fit a CDS tool into clinical workflow and identify necessary services and roles in which to integrate a CDS.

## 2. Materials and Methods

This project was conducted in a large not-for-profit healthcare system in California. As part of our focus on the principles of HCD/UCD, we adopted ideas from the “Jobs To Be Done” (JTBD) approach to product development [18]. This approach stresses the importance of understanding the specific “jobs” (e.g., calculating a patient’s CVD risk) that a user (e.g., a physician treating a patient with CVD symptoms) is trying to complete [19]. The JTBD approach also seeks to understand the benefits and limitations of the existing products or tools (e.g., an online risk calculator) to a user to complete a task. The JTBD approach is based on the belief that users ultimately are only motivated to use a new tool that is customized to the jobs they are trying to get done. In a healthcare setting, we hypothesized that if we can understand the key jobs involved in clinical decisions and execution of primary CVD prevention and the limitations and benefits of existing tools, we could then collaboratively design a digital tool that is more likely to be integrated into real-world practice.

The project team consisting of healthcare researchers and primary care physicians developed an online self-administered anonymous survey and semi-structured interview guide. Survey questions were designed to capture current CVD identification and prevention approaches, challenges in existing electronic health record (EHR) tools, and clinical “jobs” in CVD prevention (See Protocol S1: CVD Prevention PC Survey). The interview protocol was designed by project staff to capture clinician perspectives on the tasks and roles needed for effective CVD care management, the challenges faced, existing tools used in CVD preventive care, and preferences for a point-of-care health data visual display to support CVD prevention (See Protocol S2: CVD Prevention Interview). Both the survey and interview guide were reviewed by two primary care clinicians from the project team for face validity. Our hypothesis was that by identifying who performed CVD prevention, when it was performed, and what challenges were present, we could gain insight on who might be the audience for a CDS tool, where a CDS tool might be incorporated into the workflow, and what information it should include. We used convenience sampling for the survey. We contacted the CEOs of seven medical groups across our health system and received approval to put the anonymous survey in one medical group newsletter (N = 323). The survey appeared three times in the newsletter. We also received approval from one medical group to directly email each clinician’s work email (number of PCPs = 37). Participants for the email were identified using an electronic record pull of clinicians with a high proportion (75th percentile) of CVD at-risk patients in their panel. Clinicians were sent two follow-up reminder emails. Surveys were administered using REDCap Version 14 (Nashville, TN, USA). Descriptive statistics were used to analyze the survey data; a chi-square test was used to compare physician versus non-physicians, and an exact test was applied if any cell count was less than 10. These post hoc comparisons between physicians and non-physicians likely reflect their differing roles and levels of clinical responsibility in the delivery of preventive care. Consequently, statistically significant differences should be viewed as representing role-dependent perceptions of barriers or facilitators in delivering preventive care. Missing values in the surveys were not included in the analysis. A statistical analyst conducted the analysis in SAS Version 9.0 ( Cary, NC, USA).

The semi-structured interview was intended to provide more context for the survey results and was conducted after the survey. We used a purposive approach to select clinicians for the interview. An analysis of EHR data identified the top 20 primary care clinicians from two geographic regions (10 from the Bay and separately 10 from the Valley area), across the health system who had the most at-risk for CVD patients on their panel. The clinicians identified through this process were invited to participate via an initial invitation email and received up to two follow-up emails. Due to the low response, additional clinicians who treated patients at risk for CVD were selected and invited based on past CDS user experience with the project team [20]. A phone or video interview based on clinician preference was scheduled with clinicians who agreed to participate. Two project staff conducted the interview, with one staff member leading and the other taking notes. The interviews were recorded and professionally transcribed. A codebook was developed using thematic analysis and the main question categories of the interview guide as well as emergent themes. Three project staff coded the interviews using Dedoose version 9.0.107 (Hermosa Beach, CA, USA). Thirty percent of the interviews were double coded to ensure interrater reliability. The coding consistency was accessed using a Dedoose Code Application Test performed by the three coders and resulted in a pooled kappa of 0.7187. Disagreements between reviewers were discussed between the three reviewers and agreed upon by consensus. The three project staff each analyzed transcripts for the main interview themes, coded the transcripts, and summarized the findings. The project staff discussed the results, reviewed them alongside the survey results, and agreed on themes based on the codebook and emerging subthemes. The themes were shared and discussed with all authors.

The survey and interview guides and procedures were reviewed by the health system Institutional Review Board and determined to be quality improvement.

## 3. Results

Sixty-two (17%) of approximately 360 primary care clinicians (323 received the survey through a clinician newsletter, and 37 received an email containing the survey) responded to the survey. Most survey respondents were physicians (N = 47; 75.8%), in addition to nine (14.5%) physician assistants and six (9.7%) nurse practitioners. Most survey respondents had ≥20 years of clinical practice (43.5% and 42.9%, respectively) (See Table 1).

Seven out of 17 invited clinicians participated in a semi-structured interview. The interview respondents were primary care physicians (PCPs) (N = 6; 85.7%) and a nurse practitioner (N = 1, 14.3%).

Qualitative analysis of the clinician interviews identified three main themes. The findings for each theme are presented alongside the relevant survey results.

### 3.1. Identification of Patients at Risk of Developing CVD and Physician–Patient Communication

As shown in Table 2, most survey respondents (88.7%) felt that PCPs were responsible for the primary prevention of CVD, followed by patients themselves (66.1%). The interviewed clinicians echoed the survey results in that the responsibility to identify patients at risk for CVD falls on PCPs, noting that “identifying patients at risk occurs all the time as a PCP,” because they are the first to interact with patients in the healthcare system.

The survey respondents reported delivering CVD preventive care “often” during annual wellness visits (72.7%) and regular office visits (58.2%), and 40.7% reported “never”, deferring to another team for preventive care outreach. Clinicians in interviews described looking at family and health history, lifestyle, and labs to identify patients at risk of developing CVD and noted that the identification of CVD risk takes place during office and new patient visits, annual checks (similarly identified in the surveys), and when communicating about lab results.

When asked how often certain actions were taken once patients with elevated CVD risk were identified, most survey respondents (83.9%) reported “often” providing lifestyle counseling to patients focused on healthy eating, physical activity and/or smoking cessation (if relevant), and prescribing pharmacotherapy (56.4%). Half (51.8%) of respondents reported “sometimes” referring patients to a specialist, such as an endocrinologist or cardiologist, and over half (53.6%) of survey respondents responded “sometimes” referring patients to a dietitian/nutritionist or health education program. Consistent with the surveys, interviewed clinicians’ use of referrals to specialists, including a preventive cardiology program, varied and were usually infrequent. Most interviewed participants acknowledged they would refer only after they could not get a patient’s risk under control or if the patient requested to see a specialist. An interviewed clinician described referrals the following way:

*“… primary prevention, this is something I feel is bread and butter for a primary care physician to do. And really if ever I think they need a referral for a specialist, or if they refer out, it’s usually more for supportive care for them. Like nutrition. Appropriate diabetes education. Lifestyle coaching. Linking up with community resources. That type of stuff.”*

In interviews, clinicians described monitoring and communication about CVD risk during a visit being dependent on several factors including the need to manage other concerns, timing of the visit, the total time with the patient, and the nature of the conversation with the patient. Clinicians’ interview feedback reflected the ad hoc nature of identification and communication of CVD risk, noting that discussion of CVD risk could happen whenever the patient comes in and time permits.

As shown in Table 3, the majority (64.3%) of survey respondents said they regularly used the 10-year risk calculator in the EHR. Slightly under half of respondents (49.1%) reported being aware of the existence of tools in the EHR to support CVD preventive care, with only 28.5% of those aware of tools reporting that the tools were “easy to find.” In interviews, clinicians also described being aware of and able to access the CVD risk calculator and to use the risk calculator to conduct patient education, communicate risk, and determine a treatment plan. Aside from the CVD risk calculator, clinicians described using other tools, such as CHA2DS2-VASc (A-fib), the FM-IM toolkit, guidelines from US Preventive Services Task Force recommendations, American Heart Association criteria, EPIC Care Gaps, and EPIC Sticky Notes to identify patients at risk.

### 3.2. Challenges and Barriers to Delivering Preventive Care

As shown in Table 4, limited clinic time (74.2%), lack of patient engagement (51.6%), the cost of lifestyle education programs (58.1%), and lack of familiarity with lifestyle education programs (50%) were reported in surveys as making CVD preventive care difficult in a primary care setting. One surveyed clinician wrote

*“The biggest limiting factor is time. It is unreasonable for primary care to carry this burden alone. We need help. Specialty partners need to be engaged. Also patient education resources need to be available and affordable. I can’t take care of all their diagnoses plus education in a 20 min appointment.”*

Clinicians in interviews also described that PCPs do not have the bandwidth or schedule availability to prioritize preventative measures and instead act reactively when things arise. One clinician noted

*“A lot of times, patients are in, and they have a list of things that they need to talk about. And, so, sometimes, depending on how long our visits are, I may just not have the opportunity to sit back and be able to review some of those [preventive] things.”*

Additional barriers emerging in interviews included a lack of access to education programs, lack of awareness of available lifestyle programs, patient adherence to treatment plans, and the difficult nature of lifestyle changes. One clinician described the adherence problem in the following way:

*“I would say adherence is another problem. You say to someone, I really want to see you back in two weeks… And then they just don’t make the appointment, and you have no idea. I see this all the time in reviewing colleagues’ charts, too. They say they increased the blood pressure medicine. They say, come back in three months, and there’s no appointment. And, then, six months later, I’m covering their in-basket, and I get a refill request for their medication. I’m like, but they haven’t been seen. They were supposed to be seen three months ago. There’s no tracking of that at all. None.”*

In terms of follow-up visits, schedule availability and resource constraints were described as causes of not making prevention a major focus. Misalignment with specialists on the risk stratification approach (e.g., high Lp(a) versus a low CVD risk calculator score) was also highlighted as a challenge by primary care. Interviewed clinicians described a lack of straightforward processes within the EHR that would allow clinicians to communicate on the care continuum between different services, such as lifestyle counseling and cardiologist referral for patients with CVD risk. A surveyed clinician wrote about the need to standardize CVD prevention:

*“The more systematic and consistent this can be the better. Work in primary care is often chaotic and the easier and simpler something like this is to use the more likely it is to be useful and effective.”*

### 3.3. Preventive Strategies and Emerging Needs in Delivering CVD Preventive Care

Most (73.7%) survey respondents answered positively that new tools (e.g., CDS, EPIC features, dashboard reports, etc.) were needed to support primary CVD prevention (Table 3). The three most selected strategies or tools that would help deliver primary prevention of CVD were ‘tools that make referrals to lifestyle education programs easier’ (72.6%), ‘increased access to healthcare system specific CVD prevention programs for patients’ (66.1%), and ‘increased access to patient education materials about lifestyle change’ (62.9%) (Table 5).

Clinicians noted that framing lifestyle changes as investments could help encourage patients to comply. One interviewed clinician noted how it could take multiple discussions with patients before they made a needed change:

*“I really firmly believe that I see this as a journey with patients. If [a] patient is not ready for something more intense, then you got to meet them where they are. … Some people are willing to do things right away. Some people may take months. Or sometimes maybe a couple of years before they get there.”*

The ability to proactively reach out to patients was also a method for prevention that emerged during interviews. One clinician described the following:

*“… I had that really great encounter … and how to send myself a [follow up] message like, you were supposed to email me. You did not email me. And it totally fell off my radar… maybe that’s leveraging the team more, leveraging someone on the outside. It doesn’t have to be my poor, overworked medical assistant and RN that everybody’s on a shortage.”*

Clinicians described needing a way to prioritize patients for outreach and ask questions like:

*“Who are my patients that haven’t had an annual [wellness visit] ?… Or, “hasn’t been seen in more than two years.” And if you could combine that with ASCVD from their last visit two years ago, that shorter list of patients I haven’t seen that are… already identified as high risk, or patients I haven’t seen and haven’t been screened through whatever risk we want to prioritize.”*

This proactive outreach could involve leveraging analytic resources and/or different teams dedicated to identifying patients at risk and providing outreach (e.g., insurance companies contacting clinicians if patients have not filled medications, population health reaching out to chronic patients for monitoring, or medical assistants flagging patients while doing social history during an encounter).

## 4. Discussion

Our findings highlight that front line clinicians continue to face challenges in delivering preventive care services. These challenges include the limited clinical time, lack of an effective CDS tool integrating guideline-directed therapy, lack of a preventive care program and referral to specialties, limited team-based work and communication, and fragmented services around primary care. There is no systematic approach for patient risk factor identification and a lack of integrated information on downstream available services (e.g., dietitian, lifestyle counselling, preventive cardiology services) to make referrals easy. These challenges exist in part because preventive care involves multiple stakeholders and care transitions, which should also consider patients’ expectations and motivations for change.

Understanding the motivations of all stakeholders is key for engagement, behavior change, and shared decision making [21]. Motivation is affected by a wide range of human emotions and values and can be a key barrier to cardiovascular health [16]. As described in the clinician interviews and as reported in Duffy et al., understanding patient’s values and priorities may progress clinician–patient preventive communications [22]. Developing clinician–patient communication that moves beyond education and addresses patient needs and values to enhance motivation has the potential to better support effective prevention [22,23]. A CDS can help enhance communication but also must “fit” at the right time and place into the busy workflow of a clinician and readiness of the patient.

Our findings highlighted the fragmented nature of lifestyle education services within healthcare systems, where programs are often developed locally, resulting in uneven availability, limited visibility, and inconsistent referral pathways across practices and geographic regions [24]. Clinicians frequently reported a lack of awareness of available lifestyle resources and difficulties connecting patients to appropriate programs, despite strong interest in referral support. Those gaps are particularly pronounced in rural and underserved areas, where access to preventive services and lifestyle education programs is often limited [25]. Because many lifestyle interventions can be delivered virtually, a centralized CDS system implemented across all primary care practices could serve as a hub to catalog available services, standardize referral processes, and connect patients to both local and virtual programs. Such an approach has the potential to reduce geographic disparities, improve care coordination, and promote equitable access to evidence-based preventive interventions.

As described by our primary care clinicians, use of a CDS cannot rely on one stakeholder. As noted by Wang and Lo, a team-based model can facilitate bringing patients into care and support patients with downstream care, such as collecting labs, and referring patients to appropriate specialty care [26]. The mention of patients’ responsibility, referrals, and supportive care from our surveys and interviews suggests that tools and the workflow supporting the patient journey are a dominant concern (i.e., we know what needs to be done, but we do not know how to do it reliably.) The features of a CDS are important but require availability and connection among wraparound services that support clinicians and patients. Primary care clinicians play a critical role in patient identification and initiate conversations on evidence-based clinical recommendations; however, effective primary prevention cannot merely rely on primary care clinicians. A team-based model, augmented by a CDS tool, can strongly reduce a physician’s clinical burden and improve clinical efficiency and patient experience [27,28,29,30]. A next-level of team-based care model, including mapping multidisciplinary teams, clinical pharmacists, behavioral health professionals, and population health management into the patient care process [27,31] has been shown to be effective in managing high blood pressure and CVD risks [32]. Designing processes and tools that make it easier to manage referrals and coordinate with other members of the health system to address CVD prevention is needed. A team supporting PCPs who can identify and reach out to patients could help make CVD prevention and management more proactive rather than reactive. Each key stakeholder, i.e., patients, clinicians, and the healthcare system (e.g., support staff, processes), need to form communication channels where information is shared, and if acted on, shared back across teams to reduce redundancy and improve efficiency. Our results help explain that clinician-centered CDS alone may be insufficient because willing clinicians, armed with tools to identify and make recommendations, may not have the adequate related clinical resources (e.g., lifestyle consultant) necessary to deliver preventive care effectively.

Regardless of the care model, our work also highlighted the importance of the communication between the doctor and patient. Instead of relying solely on clinicians to initiate risk screening and management, patients can also play a more active role. For example, targeted educational material can be shared before a visit so that patients can initiate and/or more actively participate in a discussion of CVD risk, enhancing shared decision-making with their clinician. Therefore, future work should focus on the integration of digital tools (within or outside the EHR) to support communication before and after a visit. This will require understanding the preferred and effective communication modalities for both patients and clinicians and monitoring the communication flow. A static CDS that solely focuses on guidelines and recommendations for downstream clinical decision-making may not be sufficient; a dynamic artificial intelligence-powered CDS that covers peri-visit clinical team communication, communication with patients, and connection to support services could help shape the future of care.

A centralized CDS embedded within a team-based primary care model can serve as the operational hub that connects clinicians, patients, clinical guidelines, and preventive services, but factors such as broader policy, insurance coverage, and socioeconomic barriers, beyond CDS, must also be addressed broadly or individually with patients to achieve preventive care goals.

### Limitations

A key limitation of this project is that only a small number of clinicians were interviewed, which may not be fully representative of all perspectives on CVD preventive care delivery. We had a low response rate for interviews and did not formally assess interview theme saturation. We conducted as many interviews as we could among those clinicians who responded to the invitation. The interviews were intended to complement the survey collected from a broader primary care clinician population and were intended to be more illustrative than generalizable. The survey itself represents less than a quarter of all potential clinicians in our sample and may not be generalizable. The survey was anonymous, so we cannot compare the characteristics and representation of the non-respondents. Some of the interviewed clinicians had prior relationships with the project team from prior work, and so, they may have been biased in their views towards support tools and prevention. However, the insights from these clinicians, who were usually highly experienced with digital tools or had in-depth understanding of the barriers in delivering preventive care, can be highly informative for developing future CDS tools. The survey and interview protocol were not formally tested for content validity and may not comprehensively assess perceptions toward CVD prevention and management. However, the instruments were developed through stakeholder input and piloted for face validity. This project only gathered feedback from a clinician perspective, and we acknowledge that adding a patient perspective would provide more context, insight, and understanding of the barriers, facilitators, and expectations around the issues of clinician–patient communication expressed in the interviews. This project was exploratory in nature and from one health system, and therefore, conclusions may not be transferable to other institutions. Despite these limitations, the perspectives shared offer insight into current challenges and ways to improve CVD prevention and support the existing literature in this area.

## 5. Conclusions

CVD prevention is an important part of primary care; however, challenges around limited time and lack of resources and support make CVD preventive care inconsistent and less rigorous in practice. Preventive care should include motivating patients to make lifestyle changes, along with a coordinated effort of the health system to identify, support, and monitor patients’ health. Team-based models and digital tools are two promising directions to improve preventive care quality. Clinician feedback highlights the need to improve communication among key stakeholders (engaged patients, physicians who can act on follow up data, and health systems who can identify at-risk patients and provide additional support resources) and a need for standardized and personalized digital tools. Efforts cannot occur in isolation; coordinated work across patients, clinicians, and the healthcare system is essential for effective CVD prevention. Future work must consider the motivations of all stakeholders and how to integrate preventive CDS into workflows (1) proactively, since the current process is ad hoc, (2) efficiently, due to clinicians limited time, (3) seamlessly, to leverage existing tools, and (4) easily, to address pain points in referrals to the vital resources needed to support patients in successful preventive care.

## Figures and Tables

**Table 1:** Surveyed clinician characteristics.

Variable	Overall N = 62	Non-Physician N = 15	Physician N = 47	*p*-Value
	N (%)	N (%)	N (%)	
				0.004 ^1^
Nurse Practitioner	6 (9.7%)	6 (40%)	0 (0%)	
Physician	47(75.8%)	0 (0%)	47 (100%)	
Physician Assistant	9 (14.5%)	9 (60%)	0 (0%)	
**Years of Clinical practice**				0.06 ^1^
>=20 years	26 (43.3%)	6 (42.9%)	20 (43.5%)	
10 to < 20 years	14 (23.3%)	4 (28.6%)	10 (21.7%)	
<10 years	14 (33.4%)	4 (28.5%)	16 (34.8%)	
**Years of Using Electronic Health Records**				0.101
10+ years	41 (68.3%)	9 (64.2%)	32 (69.5%)	
<10 years	19 (31.7%)	5 (35.8%)	14 (30.5%)	

^1^ Chi-square test was used.

**Table 2:** Current CVD practices survey response.

Response Options	Overall N = 62	Non-Physician N = 15	Physician N = 47	*p*-Value
**In your opinion, which of the following roles should be primarily responsible for primary prevention of CVD?**				
Primary Care Physician	55 (88.7%)	13 (86.7%)	42 (89.4%)	0.77 ^1^
Nurse Practitioner/ Physician Assistant	34 (54.8%)	14 (93.3%)	20 (42.6%)	0.0006 ^1^
Case Manager	13 (21.0%)	5 (33.3%)	8 (17%)	0.27 ^2^
Patient	41 (66.1%)	12 (80%)	29 (61.7%)	0.19 ^1^
Other	3 (4.8%)	1 (6.7%)	2 (4.3%)	1.0 ^2^
**How do you usually deliver primary prevention of CVD**?				
**Annual physical examination/wellness visits**				0.17 ^2^
Often	40 (72.7%)	8 (57.1%)	32 (78%)	
Sometimes	15 (27.3%)	6 (42.9%)	9 (22%)	
**Regular office visits**				0.12 ^1^
Often	32 (58.2%)	7 (50%)	25 (61%)	
Sometimes or Rarely	23 (41.8%)	7 (50%)	16 (39.0%)	
**Virtual visits (video visit/telephone visit)**				0.23 ^2^
Often	13 (23.6%)	2 (14.3%)	11 (26.8%)	
Sometimes or Rarely	42 (76.3%)	12 (85.7%)	22 (73.2%)	
**When reminded by a Medical Assistant (MA) or other member of our office care team**				0.27 ^1^
Often or Sometimes	16 (30.2%)	4 (33.3%)	12 (29.3%)	
Rarely or Never	37 (69.8%)	8 (66.7%)	18 (70.7%)	
**MHO message**				0.08 ^1^
Often or Sometimes	25 (47.1%)	7 (58.4%)	18 (43.9%)	
Rarely or Never	28 (52.9%)	5 (41.6%)	23 (56.1%)	
**Defer to another team for outreach**				0.14 ^2^
Sometimes	11 (20.4%)	4 (33.3%)	7 (16.7%)	
Rarely or Never	43 (79.6%)	8 (66.7%)	35 (83.3%)	
**Provide lifestyle counseling on healthy eating, physical activity and/or smoking cessation (if the patient is a smoker)**				0.68 ^1^
Often	47 (83.9%)	11 (78.6%)	36 (85.7%)	
Sometimes	9 (16.1%)	3 (21.4%)	6 (14.3%)	
**If you have a patient at risk of developing CVD, how often do you do the following**				
**Refer to a dietitian/nutritionist or health education programs**				0.34 ^1^
Often	6 (10.7%)	3 (21.4%)	3 (7.1%)	
Sometimes	30 (53.6%)	7 (50%)	23 (54.8%)	
Rarely	20 (35.7%)	4 (28.6%)	16 (38.1%)	
**Prescribe pharmacotherapy**				0.63 ^2^
Often	31 (56.4%)	6 (46.2%)	25 (59.5%)	
Sometimes	23 (41.8%)	7 (53.8%)	16 (38.1%)	
Rarely/Never	1 (1.8%)	0 (0%)	1 (2.4%)	
**Refer to a specialist (e.g., endocrinologist, cardiologist)**				0.03 ^1^
Often	5 (8.9%)	3 (21.4%)	2 (4.8%)	
Sometimes	29 (51.8%)	9 (64.3%)	20 (47.6%)	
Rarely/Never	22 (39.2%)	2 (14.2%)	20 (47.6%)	

^1^ Chi-square test was used. ^2^ Fisher exact test was used.

**Table 3:** 10 year CVD risk calculator survey responses results.

Variable	Overall N = 62	Non-Physician N = 15	Physician N = 47	*p* Value
	N (%)	N (%)	N (%)	
**Existence of CDS tool(s) in the EHR**				0.25 ^1^
Yes	28 (49.1%)	5 (35.7%)	23 (53.5%)	
No	29 (50.9%)	9 (64.3%)	20 (46.5%)	
**New CDS tool(s) for CVD preventive care are needed?**				0.74 ^2^
Yes	42 (73.7%)	11 (78.6%)	31 (72.1%)	
No	15 (26.3%)	3 (21.4%)	12 (27.9%)	
**Regular use 10-year CVD risk**				0.20 ^1^
Yes	36 (64.3%)	7 (50%)	29 (69%)	
No	20 (35.7%)	7 (50%)	13 (31%)	
**What do you use the risk calculation for?**				
To decide on antihypertensive treatment	8 (12.9%)	3 (20.0%)	5 (10.6%)	0.39 ^2^
To decide on lipid lowering treatment	36 (58.1%)	7 (46.7%)	29 (61.7%)	0.30 ^1^
To decide on diabetes treatment	4 (6.5%)	0 (0%)	4 (8.5%)	0.56 ^2^
To decide on obesity/overweight treatment	4 (6.5%)	1 (6.7%)	3 (6.4%)	1.0 ^2^
To decide on lifestyle advice	15 (24.2%)	3 (20.0%)	12 (25.5%)	1.0 ^2^
**Reasons not to use the 10-year CVD risk calculator?**				
Limited time	32 (51.6%)	8 (53.3%)	24 (51.1%)	0.88 ^1^
Not useful for treatment decisions	4 (6.5%)	3 (20.0%)	1 (2.1%)	0.04 ^2^
Do not know where to find the calculator	12 (19.4%)	4 (26.7%)	8 (17%)	0.46 ^2^
Do not know how to use/interpret the risk score	4 (6.5%)	2 (13.3%)	2 (4.3%)	0.24 ^2^
Inconsistency among different guidelines	7 (11.3%)	1 (6.7%)	6 (12.8%)	1.0 ^2^
Other	15 (24.2%)	2 (13.3%)	13 (27.7%)	0.32 ^2^

^1^ Chi-square test was used. ^2^ Fisher exact test was used.

**Table 4:** Current challenges responses from survey.

Response Options	Overall N = 62	Non-Physician N = 15	Physician N = 47	*p*-Value
**What are the main challenges that make delivering CVD prevention difficult in a primary care setting?**				
Limited clinic time with patient	46 (74.2%)	10 (66.7%)	36 (76.6%)	0.44 ^1^
Not able to identify patients who would benefit from primary prevention	9 (14.5%)	1 (6.7%)	8 (17.0%)	0.43 ^2^
Lack of support staff	20 (32.3%)	4 (26.7%)	16 (34%)	0.75 ^2^
Lack of patient engagement	32 (51.6%)	9 (60.0%)	23 (48.9%)	0.46 ^1^
Not covered by patient’s insurance	18 (29.0%)	5 (33.3%)	13 (27.7%)	0.75 ^2^
Not a system/department priority	7 (11.3%)	1 (6.7%)	6 (12.8%)	1.0 ^2^
Not familiar with lifestyle education programs provided in the healthcare	31 (50.0%)	9 (60.0%)	22 (46.8%)	0.37 ^1^
Cost of lifestyle education programs	36 (58.1%)	10 (66.7%)	26 (55.3%)	0.44 ^1^
Guidelines change constantly	18 (29.0%)	3 (20%)	15 (31.9%)	0.52 ^2^

^1^ Chi-square test was used. ^2^ Fisher exact test was used.

**Table 5:** Tools and prevention strategies survey responses.

Variable	Overall N = 62	Non-Physician N = 15	Physician N = 47	*p* Value
**Strategies or tools useful in primary prevention of CVD (SELECT ALL THAT APPLY)**				
Tools that educate provider on primary prevention of CVD	22 (35.5%)	5 (33.3%)	17 (36.2%)	0.84 ^1^
Tools that provide up-to-date primary prevention of CVD guidelines	38 (61.3%)	12 (80%)	26 (55.3%)	0.09 ^1^
To help identify at risk patients and make recommendations about primary prevention of CVD	34 (54.8%)	9 (60.0%)	25 (53.2%)	0.64 ^1^
Tools that make referrals to lifestyle education programs easier	45 (72.6%)	12 (80%)	33 (70.2%)	0.53 ^1^
Increased access to patient education materials about lifestyle change	39 (62.9%)	11 (73.3%)	28 (59.6%)	0.34 ^1^
Increased access to Sutter specific CVD prevention programs for patients	41 (66.1%)	12 (80%)	29 (61.7%)	0.19 ^1^

^1^ Chi-square test was used.

## Data Availability

De-identified data are available on request only upon complying with our institute’s privacy and legal requirements.

## Supplementary Materials

The following supporting information can be downloaded at: https://www.mdpi.com/article/10.3390/healthcare14182937/s1, Protocol S1: CVD Prevention PC Survey; Protocol S2: CVD Prevention Interview.

## Abbreviations

The following abbreviations are used in this manuscript:

CVD	Cardiovascular Disease
CDS	Clinical Decision Support
EHR	Electronic Health Record
HCD	Human-Centered Design
JTBD	Jobs To Be Done
PCP	Primary Care Physicians
UCD	User-Centered Design
