# Supplementary material for: User-Centered Design Approach to Dynamic Clinical Decision Support in Primary Prevention of Cardiovascular Disease
Source: Healthcare (Basel). 2026 Sep 10;14(18):2937. doi: 10.3390/healthcare14182937 (PMC13606592; doi:10.3390/healthcare14182937)
Supplement: Supplementary file 1 [file healthcare-14-02937-s001.zip › Protocol S1-CVD Prevention PC Survey.pdf]

Primary Cardiovascular Disease Prevention Survey: Perceptions and Practices

Introduction

This one-time, 5-minute, quality improvement survey is part of an NIH-funded project focused on preventive care. The purpose of the survey is to identify ways to reduce the burden of CVD preventive care on clinicians. Despite the importance of preventive care, it can be difficult to provide these services in an ambulatory environment where encounters are short and focused on acute needs, administrative burdens are high, and payment models may not support preventive care services. The results of this survey will be used to better understand facilitators and barriers to primary CVD prevention and what resources are used or recommended to patients for prevention and/or treatment.

Completing this survey is optional. All survey data will be kept in a password-protected environment behind the Sutter Health firewall. Email addresses are used for invitation purposes only and will not intentionally be linked to survey answers.

Questions

1. **I am a:**
    a. Physician
    b. Physician Assistant
    c. Nurse Practitioner
    d. Other
    If you selected other, please specify: ________________________

2. **Please specify how many years of experience you have in the following:**

	**<5 years**	**5 to <10 years**	**10 to <20 years**	**≥20 years**
a. Clinical practice in general				
b. Clinical practice at Sutter Health				
c. Using Electronic Health Records				

3. **In which Sutter Health foundation do you primarily practice?**
    a. Palo Alto Medical Foundation (PAMF)
    b. Sutter East Bay Medical Foundation (SEBMF)
    c. Sutter Pacific Medical Foundation (SPMF)
    d. Sutter-Gould Medical Foundation (SGMF)
    e. Sutter Medical Foundation (SMF)
    f. Sutter Medical Group of the Redwoods (SMGR)
    g. Sutter Independent Physicians (SIP)
    h. Other
    If you selected other, please specify: ________________________

4. **In your opinion, which of the following roles should be primarily responsible for primary prevention of CVD? (SELECT ALL THAT APPLY)**
    a. Primary Care Physician
    b. Nurse Practitioner/Physician Assistant
    c. Case Manager
    d. Patient
    e. Other
    If you selected other, please specify: __________

5. **What are the main challenges that make delivering CVD prevention difficult in a primary care setting? (SELECT ALL THAT APPLY)**
    a. Limited clinic time with patient
    b. Not able to identify patients who would benefit from primary prevention
    c. Lack of support staff
    d. Lack of patient engagement
    e. Not covered by patient's insurance
    f. Not a system/department priority
    g. Not familiar with lifestyle education programs provided at Sutter
    h. Cost of lifestyle education programs
    i. Guidelines change constantly
    j. Other
    If you selected other, please specify: __________

The following questions are related to primary CVD prevention tools available to you (e.g. tools in the EHR that help you identify patients for CVD prevention, treatment, or referral etc.) at Sutter Health to support primary prevention for CVD.

6. **Are you aware of the existence of tool(s) in the EHR to support primary prevention of CVD?**
    a. Yes
    b. No

If answer to Q6 is "No" skip to Q8, if "Yes" ask Q7

7. **Can you tell us about your experience of existing Sutter Health tool(s), e.g. clinical decision support tools and SmartSet, to support primary prevention of CVD?**

	**Strongly agree**	**Agree**	**Neutral**	**Disagree**	**Strongly disagree**	**N/A**
Tools are easy to find						
Tools are easy to use						
Tools are easy to interpret						
Tools are easy to act on						

8. **Do you believe that new tool(s) (e.g., clinical decision support, EPIC features, dashboard reports, etc.) to support primary prevention of CVD are needed?**
    a. Yes
    b. No

The next questions ask about how you routinely practice primary prevention of CVD.

9. **Do you regularly use the 10-year CVD risk calculator in EPIC for patients aged 40-70 years old?**
    a. Yes
    b. No

If answer to Q9 is "No" skip to Q11, if "Yes" ask Q10

10. **What do you use the risk calculation for? (SELECT ALL THAT APPLY)**
    a. To decide on antihypertensive treatment
    b. To decide on lipid lowering treatment
    c. To decide on diabetes treatment
    d. To decide on obesity/overweight treatment
    e. To decide on lifestyle advice
    f. Other
    If you selected other, please specify: __________

11. **What are the reasons you may not use the 10-year CVD risk calculator when managing CVD risk reduction? (SELECT ALL THAT APPLY)**
    a. Limited time
    b. Not useful for treatment decisions
    c. Don't know where to find the calculator
    d. Don't know how to use/interpret the risk score
    e. Inconsistency among different guidelines
    f. Other
    If you selected other, please specify: __________

12. **If you have a patient at risk of developing CVD, how often do you do the following?**

	Never	Rarely	Sometimes	Often
a. Provide lifestyle counseling on healthy eating, physical activity and/or smoking cessation (if the patient is a smoker)				
b. Refer to a dietitian/ nutritionist or health education programs				
c. Prescribe pharmacotherapy				
d. Refer to a specialist (e.g. endocrinologist, cardiologist).				

13. **What <u>strategies or tools</u> would help you in primary prevention of CVD?** (**SELECT ALL THAT APPLY**)
    a. Tools that educate provider on primary prevention of CVD
    b. Tools that provide up-to-date primary prevention of CVD guidelines
    c. Clinical decision support tools to help identify at risk patients and make recommendations about primary prevention of CVD
    d. Tools that make referrals to lifestyle education programs easier
    e. Increased access to patient education materials about lifestyle change to promote provider-patient conversation on CVD prevention
    f. Increased access to Sutter specific CVD prevention programs for patients
    g. Other
    If you selected other, please specify: __________

14. **How do you usually deliver primary prevention of CVD?**

	Never	Rarely	Sometimes	Often
a. Annual physical examination/wellness visits				
b. Regular office visits				
c. Virtual visits (video visit/telephone visit)				
d. When reminded by a MA or other member of our office care team				

e.	MHO message				
f.	Defer to another team for outreach				

15. **If you have additional comments on CVD prevention, please write them here:** *(not a required question)*
