# Supplementary material for: User-Centered Design Approach to Dynamic Clinical Decision Support in Primary Prevention of Cardiovascular Disease
Source: Healthcare (Basel). 2026 Sep 10;14(18):2937. doi: 10.3390/healthcare14182937 (PMC13606592; doi:10.3390/healthcare14182937)
Supplement: Supplementary file 1 [file healthcare-14-02937-s001.zip › Protocol S2-CVD Prevention Interview.pdf]

Primary Care Clinician Interview Protocol

Study: Preventing Cardiovascular Disease (CVD) through Improved Care Coordination via Re-engineering of Electronic Health Records and Tailored Patient Communication

Sponsor: National Institute on Aging (NIA)

The following protocol is designed to be used as a guideline for interviewing clinicians who deliver preventive CVD care to patients at Sutter Health. Interviews are estimated to take 60 minutes. The purpose of this interview is to understand the current state of the CVD preventive care process, including the jobs or tasks that clinicians do to identify and treat patients at risk of developing CVD and challenges related to these tasks.

Clinician Interview Process

1. Sutter Health primary care clinicians will be asked to participate in an interview about their experience delivering preventive care to patients at risk of developing CVD. The interviewer and/or project manager will email and/or call clinicians to invite them to participate.

2. The following language will be used to invite clinicians to participate in interviews:

   We are exploring the idea of designing a solution that would support preventive cardiovascular disease (CVD) treatment at Sutter Health. We would like to interview you about the steps you take to care for patients at risk of developing CVD and your perspective on any challenges or things that could make it easier for you to deliver care.

   If you are willing to set up a 60-minute call with me, please just let me know what days and times are best for you.

3. For clinicians that are already connected with the Center for Health Systems Research (CHSR), correspondence about participating will be coordinated through the CHSR team member that they know, and the language above will be tailored to invite them to participate.

4. During interviews, the interviewer will ask for permission to record using the following language as a guide:

   I'd like to record our interview for note taking purposes to make sure that I capture all the detail you share with me. We won't be attaching anything that you say to your name and all the information you share with us will be combined with comments from other clinicians who we are interview, so this conversation is confidential. Is it okay with you if we record? The recording will be saved on a secure network drive behind the Sutter firewall. (If yes, start recording)

5. Interview questions are open ended. The interviewer will ask follow-up questions based on how clinicians respond to questions in order to explore details around why and how clinicians view their jobs, roles, challenges, etc.

Interview Date	
Interviewer Name	
Interviewee Name/Study ID	
Consent signed (Y/N)	No – not required

Clinician Interview Questions

Practice location

Role:
Location:

Introduction

Thank you for taking the time to participate in the interview. This interview will last about one hour. The purpose of this interview is to understand the current state of the CVD preventive care process. This includes the jobs or tasks that clinicians do to identify, communicate, and treat patients at risk of developing CVD, along with the challenges related to these tasks.

I'd like to record our interview for note taking purposes to make sure that I capture all the detail you share with me. We won't be attaching anything that you say to your name and all the information you share with us will be combined with comments from other clinicians we interview, so this conversation is confidential. The recording will be saved on a secure network drive behind the Sutter firewall. Is it okay with you if we record? (If yes, start recording)

Identification of patients

1. How do you identify patients at risk of developing CVD?
   a. Probe: When in your workflow do you identify patients?
   b. What tools do you use to identify patients at risk of developing CVD?
      i. What are the advantages of the tool you are currently using? Which one is the best?
      ii. Probe: In Epic, can you walk through the items you use to identify patients?
      iii. What are the challenges that you face when using the current tool?
      iv. How often do you use an ASCVD (atherosclerotic cardiovascular disease) risk estimator? Where do you find it (in EPIC or from estimator website)?

Communication of risk and risk education

Next, we're going to move onto the communication of risk and risk reduction.

1. How do you usually communicate/inform the patient of the risk of developing CVD?
    a. Probe: When in your workflow do you communicate the risks with the patient?
    b. How do you explain what risk of CVD means?
        i. Do you emphasize the current status and indicators (Lab results, BP, BMI, smoking, etc.)?
        ii. Do you emphasize the potential consequence (future stroke, MI)?
        iii. Or both?

2. What are the challenges that you face when communicating risk of developing CVD and risk reduction with the patients?
    a. Probe: Has there been anything that has helped you communicate risk of CVD and risk reduction with patients?

•

Treatment/management

1. Now we're going to talk about the decision-making process for the management and treatment of CVD primary prevention.
    a. Who is involved in the decision making?
        i. What is the role of the patient?
    b. Is there any difference in handling different CVD risk factors? (For example, obesity, DM(diabetes?), HTN?) Can you describe the decision-making process for each of the common risk factors?
    c. Is there any difference in handling of patients of different ages (for example, <65 vs. >=65 years old)?
    d. Do you usually start with referrals to education programs or consult patients with lifestyle changes yourself? Do you find it is easy to locate education programs available at Sutter?

2. How do you continue monitoring a patient at risk of developing CVD?
    a. What does follow-up care entail?
        i. Treatment targets?
    b. Who/what roles are involved? What is communication like across the care team?

3. What are the challenges that you face when monitoring a patient at risk of developing CVD?
    a. Adherence to medication/ suggested lifestyle changes
    b. Referral to specialist
    c. Referral to health education programs
    d. Frequency of visits/interaction/follow up with the patient?
    e. Communication across the care team?

4. When do you refer patients at risks of developing CVD to specialists (Cardiologist, endocrinology)?What drives your decision to refer patients to specialists?

5. What tools do you use to manage patients at risk of developing CVD?
    a. What are the advantages of that tool? Which one is the best?
        i. Probe: In Epic, can you walk through the items you use to manage patients?
    b. What are the challenges that you face when using that tool?

•

Additional Questions

1. The phases of the preventative care process that we've covered in this interview are the identification, communication, treatment, and management. Are there any other phases that you would include?
2. Where do you go to see the preventive care guidelines? Which guidelines do you usually use for CVD prevention? What are the challenges you currently face in locating/adopting guidelines for CVD prevention?
3. We're looking to recruit several pilot clinicians for our solution. As a pilot clinician, you will be asked to give insight on your workflow and provide ideas and feedback during the design process. You will also be asked to test and pilot the solution in your clinic, notifying our team of any issues or feedback you have along the way. Would you be interested in being a pilot clinician?
4. (If time allows) Are there any questions you have for me, or anything else you would like to add?

•
